# Surface-enhanced Raman scattering of self-assembled thiol monolayers and supported lipid membranes on thin anodic porous alumina

**DOI:** 10.3762/bjnano.8.8

**Published:** 2017-01-09

**Authors:** Marco Salerno, Amirreza Shayganpour, Barbara Salis, Silvia Dante

**Affiliations:** 1 Department of Nanophysics, Istituto Italiano di Tecnologia, via Morego 30, I-16163 Genova, Italy; 2 Department of Bioengineering and Robotics, University of Genova, viale Causa 13, I-16145 Genova, Italy

**Keywords:** anodic porous alumina, SERS, nanopores, supported lipid bilayers, thiols

## Abstract

Thin anodic porous alumina (tAPA) was fabricated from a 500 nm thick aluminum (Al) layer coated on silicon wafers, through single-step anodization performed in a Teflon electrochemical cell in 0.4 M aqueous phosphoric acid at 110 V. Post-fabrication etching in the same acid allowed obtaining tAPA surfaces with ≈160 nm pore diameter and ≈80 nm corresponding wall thickness to be prepared. The tAPA surfaces were made SERS-active by coating with a thin (≈25 nm) gold (Au) layer. The as obtained tAPA–Au substrates were incubated first with different thiols, namely mercaptobenzoic acid (MbA) and aminothiol (AT), and then with phospholipid vesicles of different composition to form a supported lipid bilayer (SLB). At each step, the SERS substrate functionality was assessed, demonstrating acceptable enhancement (≥100×). The chemisorption of thiols during the first step and the formation of SLB from the vesicles during the second step, were independently monitored by using a quartz crystal microbalance with dissipation monitoring (QCM-D) technique. The SLB membranes represent a simplified model system of the living cells membranes, which makes the successful observation of SERS on these films promising in view of the use of tAPA–Au substrates as a platform for the development of surface-enhanced Raman spectroscopy (SERS) biosensors on living cells. In the future, these tAPA–Au-SLB substrates will be investigated also for drug delivery of bioactive agents from the APA pores.

## Introduction

Anodic porous alumina (APA) is a layered material usually obtained in thick form (≈10 µm thickness scale) from electrochemical anodization in the acidic aqueous electrolyte of aluminum (Al) foils [1]. In APA, the control of pore size, pore density and porosity is achieved by changing the anodization voltage during the fabrication and the etching parameters during the post-fabrication treatment [2]. It is widely recognized that the APA surface is biocompatible with practically all cell types and provides a means of controlling the surface roughness [3–4], the latter of which can play an important role in the adhesion and proliferation of cells [5–7]. The self-ordered nano-structured APA, also demonstrated recently as a possible nanolithographic mask [8–9] and for chemical sensors and biosensors [10], after coating with noble metals can be used for plasmonics-based enhanced spectroscopy such as in surface-enhanced Raman spectroscopy (SERS) [11–14].

In recent years, the thin form of APA (tAPA), resulting from anodization of Al films of less than 1 µm thickness, has been increasingly used because it can be better integrated into applications involving optical microscopy inspection, which requires flat planar substrates. Moreover, it allows to move toward a more robust engineering of APA surfaces by exploiting the standard microtechnology of photolithography, thereby paving the way to large scale fabrication in possible future devices.

The enhancement factor in APA-based SERS can be as high as 1000, which means that the technique may detect molecules [15]. Additionally, the pores in tAPA can potentially serve as nano-wells for localized drug delivery [16–17]. In fact, while lower in loading capacity with respect to thick APA [18], 500 nm tAPA can still allocate a significant amount of bioactive compounds, representing a trade-off between the former case of maximized loading and the case of ultra-thin APA showing the highest SERS enhancement [19]. Finally, the controlled roughness of APA could also improve the physisorption of coating layers of functional materials [20–21].

The main component of the biological membrane that separates and protects the interior of all living cells from the outside environment is a phospholipid bilayer. For this reason, as well as for the complexity of real samples of living cells, we decided to test the tAPA–Au SERS-active substrates on SLBs in phosphate-buffered saline (PBS) buffer solution, which provide an excellent model system to mimic the native cellular membranes [22].

In the present work, the fabrication and modification of tAPA aiming at its exploitation as a functional substrate for biosensing based on SERS effect are presented. In particular, it is reported on SERS effect on SLBs obtained from spontaneous lipid vesicle fusion and representing a simplified model of living cells membrane. Since the vesicle fusion is not trivial to achieve on Au surfaces, we first functionalized the Au with self-assembled monolayers (SAM) of thiols, to provide the appropriate surface condition to allow SLB formation. SERS effect was tested and proved for each fabrication step of the system.

## Experimental

### tAPA fabrication and modification to achieve SERS-activity

An ≈500 nm thick Al layer was first coated on a silicon wafer by an electron-beam evaporation system PVD75 (Kurt J. Lesker Ltd., UK) working at a base pressure of 10^−6^ Torr with a deposition rate of 0.5–1 Å/s. tAPA was fabricated in a single-step (≈15 min) anodization performed at 110 V in 0.4 M phosphoric acid electrolyte at a bath temperature of ≈15 °C. Post-fabrication etching in the same electrolyte for 20 min at room temperature (RT) plus 15 min at 35 °C allowed to obtain tAPA with ≈160 nm pore size and ≈80 nm wall thickness. After thoroughly rinsing with de-ionized water, blowing dry with nitrogen and dehydrating on a hotplate set at 100 °C for 15 min, the tAPA was overcoated by the same electron-beam evaporation system with a ≈25 nm thick Au layer to make it SERS-active. More details on similar fabrication procedure can be found in references [12–13].

The characteristic size of tAPA pores was obtained by scanning electron microscope (SEM) imaging with a JSM-7500F (Jeol, Japan) and subsequent grain analysis carried out with Igor 6.22 (Wavemetrics, OR, USA).

### Incubation of thiols and fabrication of lipid vesicles

Different thiols were used in combination with the different lipids to be coated onto them by electrostatically-driven physisorption. We used two thiols, namely 4-mercaptobenzoic acid (MbA) and 11-amino-1-undecanethiol hydrochloride (AT), from Sigma (Milan, Italy), and three lipids, namely 1-palmitoyl-2-oleoyl-*sn*-glycero-3-phosphocholine (POPC), 1-palmitoyl-2-oleoyl-*sn*-glycero-3-phospho-L-serine (POPS) and, 1,2-dioleoyl-3-trimethylammoniumpropane (DOTAP), from Avanti Polar Lipids (Alabaster, Alabama, US). All solvents were purchased from Sigma-Aldrich.

First, the substrates were incubated at rt for 2 h with a 1 mM aqueous solution of the thiol molecule, either MbA or AT, to let the sulfur of the –SH group bind covalently to the Au surface (chemisorption). The substrates were then gently washed with their aqueous solutions and dried under nitrogen flow.

All the lipids were dissolved in chloroform/methanol 2:1 vol/vol, dried under a gentle nitrogen flux in a test tube, and put under a mild vacuum overnight to remove all solvent traces. POPC/POPS in a 9:1 mol/mol ratio and DOTAP were then re-suspended in PBS at a 5 g/L concentration, let to swell for 30 min, and extruded 11 times through a polycarbonate filter (Whatman, USA) with 100 nm pore diameter to form unilamellar vesicles.

### Preparation of the Raman target analytes: SLBs

The lipid vesicles were diluted to 0.5 g/L in the PBS buffer and vortexed immediately before use. The thiol SAM was incubated overnight with the lipid vesicle dispersion, to allow vesicle physisorption and fusion onto the substrate. The following day the samples were carefully washed with PBS three times to remove the exceeding vesicles.

For the cationic lipids (namely DOTAP), we used a thiolated molecule that presents a positively charged group at its end. This is MbA, whose COOH group is protonated in PBS buffer to COO^−^. For the anionic lipid mixture (namely POPC/POPS) AT was used, which becomes positive in aqueous solution because of the terminal amino group.

### SERS measurements

SERS measurements were performed with a micro-Raman spectrometer inVia (Renishaw, UK) equipped with the software program WiRE 3.2. We used for excitation a laser with 785 nm wavelength and 100 mW power, equipped for dispersion with a grating with 1200 grooves/mm. For detection microscope objectives with a magnification of 50× (NA: 0.75) and 60× (water immersion, NA: 1.0) were used. The spectra were collected in the 300–3200 cm^−1^ spectral range.

The SERS enhancement factor G achieved by employing tAPA–Au with respect to the flat Au on silicon substrate can be estimated by using a simple formula:

[1]
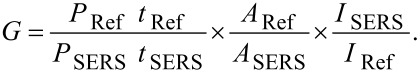


where *P*, *t*, *A* and *I* are laser power, accumulation time, active area for molecule adsorption and Raman intensity of the specific band, respectively [13]. The subscripts, SERS and Ref, indicate SERS and Raman measurements on tAPA–Au and on flat Au substrates, respectively.

### QCM-D characterization of adsorption

A quartz microbalance Z500 (KSV Instruments, Finland) was used for the QCM-D experiments. Au coated AT-cut quartz crystals (QSense, Sweden) with a 5 MHz fundamental resonance frequency were used. Before each experiment, the quartz sensor was first cleaned in a UV/Ozone ProCleaner (BioForce Nanoscience, US) for 10 min, then washed with milli-Q (18.2 MΩ·cm resistivity) water, dried under nitrogen flux and cleaned again for 10 min in the ozone cleaner.

The sensor was then mounted in the measurement chamber. The chamber was filled with proper buffer (aqueous solution for thiols, PBS for DOTAP vesicles and milli-Q water for POPC/POPS vesicles), and left to reach an equilibrium (≈30 min) before injecting the solution of interest. 3 mL of solution (1 mM thiols in aqueous solution, and a concentration of 0.25 g/L for both DOTAP in PBS and POPC/POPS in milli-Q) where then injected in the measurement chamber and left until the adsorption process reached an end (overnight measurement for thiols, 2 h for vesicles). The sensor was then rinsed with the proper buffer solution. If not stated otherwise, changes in frequency and dissipation of the seventh overtone (35 MHz) are shown; all experiments were carried out at a temperature of 22 °C.

## Results and Discussion

### SERS-active tAPA–Au substrates

**Figure 1:**
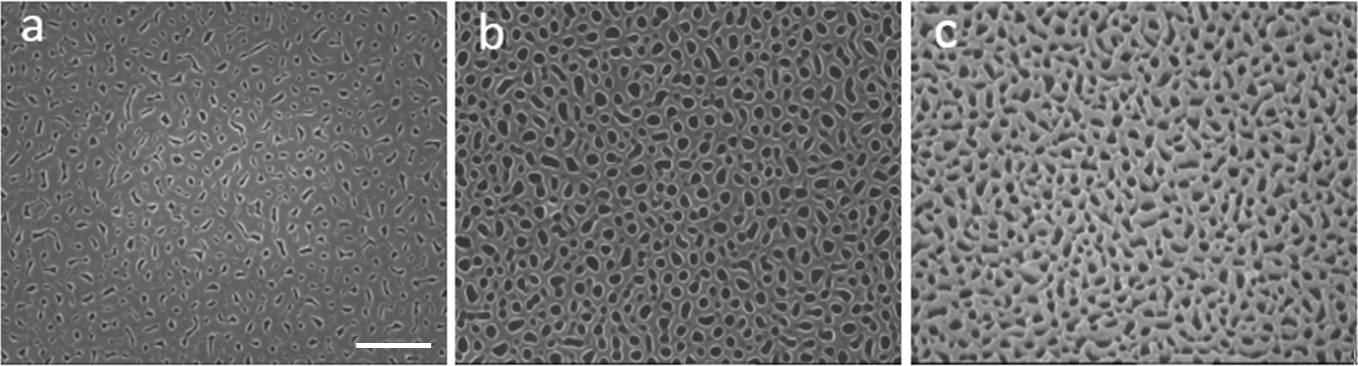
SEM images (20,000× magnification, scale bar 1 µm) of tAPA substrates (thickness ≈500 nm), a) as-prepared, b) after pore widening, and c) after 25 nm Au coating.

The control of the geometrical features of nanostructured substrates is of critical importance in SERS [23]. The SEM images reported in Figure 1 show the good control achieved in both mean pore size and its dispersion and prove the long range uniformity of the surfaces with the Au coating to make it plasmonic-active.

tAPA–Au substrates could possibly be used as a carrier layer for local drug delivery [24], as a substrate for living cell cultures thanks to its controlled porosity [3–7], and for SERS [25]. However, since SERS is a surface-only effect, this sensitive detection will be limited to the top of the tAPA–Au substrates, i.e., to the bottom of the living cells, where they would adhere to the nanoporous substrate.

### SERS enhancement due to tAPA–Au on thiols, lipids, and thiol–lipid systems

The Raman measurements were performed first on the thiol molecules. We started from the raw materials, in powder form, to obtain reference spectra for future comparison and best identification of the typical bands. Then, we measured the Raman scattering of the thiols adsorbed to flat Au substrates. For technical reasons of SLBs assembly, the two thiols selected, MbA and AT, in ethanol and PBS solutions have a negatively or positively charged group, respectively.

**Figure 2:**
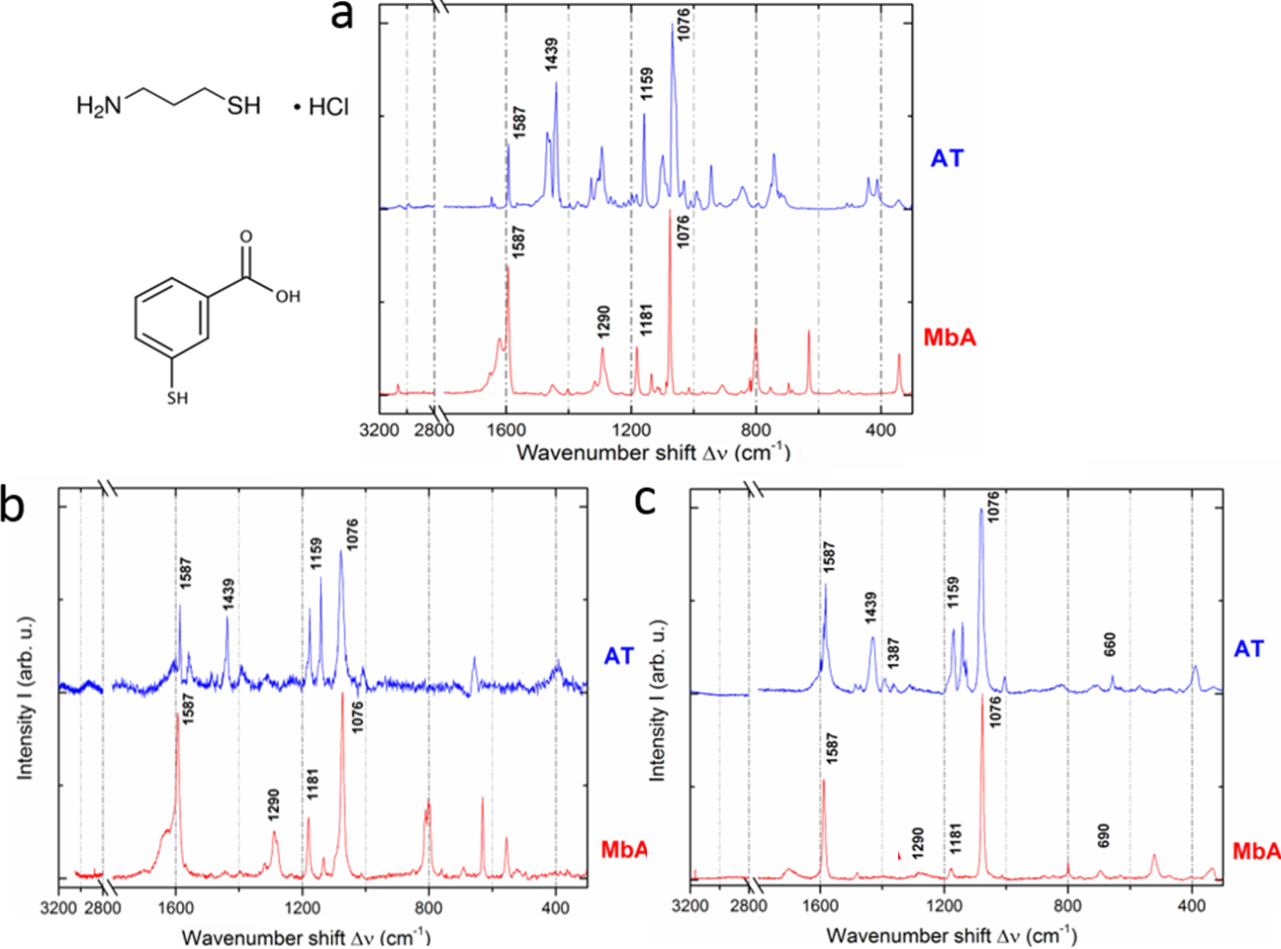
Raman spectra of thiols a) in powder form (with their molecular structures); b) in flat film form, after adsorption to the flat Au substrates from water solution; c) in film form on tAPA–Au.

The spectra of the thiols powder on flat Au, along with the respective molecular structures, are shown in Figure 2a. In Figure 2b the spectra of the SAM of the same thiols obtained after incubation on flat Au from 1 mM water solution for 2 h at RT are shown. The subsequent step was the deposition of the thiol molecules for the formation of SAM onto tAPA–Au and the observation of the respective spectra. The nanopores in the oxide under the Au, which are replicated by the top Au surface thanks to the low Au thickness of ≈25 nm, allowed for SERS effect. In Figure 2c we report the typical Raman spectra obtained on tAPA–Au for both MbA and AT.

The spectra of each thiol in all forms (pristine powder and film adsorbed onto the flat Au and tAPA–Au substrate) look similar. MbA present two major peaks at ≈1593 and ≈1076 cm^−1^, which can be ascribed to aromatic ring vibrations, and also at ≈1181 and at ≈1290 cm^−1^, which belong to C–H mode [26–28]. AT presents the major peaks at ≈1434 and ≈1477 cm^−1^ assigned to the C–H and at ≈1074 cm^−1^ assigned to the N–H, while the peak at ≈1074 cm^−1^ belongs to the C–C stretching.

In Supporting Information File 1, Figure S1 again the Raman spectra of both thiol SAMs, coupled according to the same thiol deposited on the different substrates of flat Au and tAPA–Au, are presented, for easier visualization of the substrate effect. It appears clearly that on tAPA–Au the major characteristic peaks of both MbA and AT are highly enhanced. Taking into account the measurement parameters (i.e., *t*_Ref_ and *t*_SERS_ both 10 s, while *P*_Ref_ and *P*_SERS_ are 100 and 1, respectively), a G factor of approximately 600 and 1000 was calculated for MbA and AT, respectively. The SERS effect of the nanophotonic tAPA structure, after coating with Au and thus thanks to the localized surface plasmon resonances of this thin film, emerges. The same effect may also be used on the SLBs, at the later stage of the model system fabrication.

**Figure 3:**
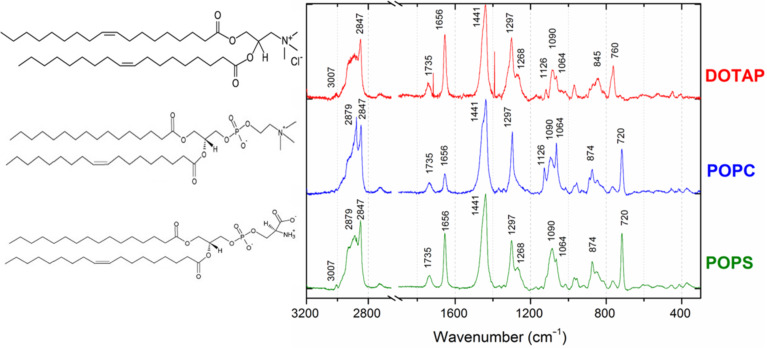
Raman spectra of lipids in powder form on flat Au substrates, with their molecular structures.

For the selected lipids, we first tested the Raman spectra of the powders and then of the SLB form, on both the flat Au and nanoporous tAPA–Au. The molecular structure and Raman spectra of lipids in powder form are shown in Figure 3.

The molecules of choice, i.e., POPC and POPS, are two glycerophospholipids largely present in real cellular membranes. In particular, we prepared a mixture of POPC and POPS suspended in PBS with the molar ratio of 8:2, in order to resemble the plasma membrane composition both for charge and acyl chain length and unsaturation grade. However, the reason for the choice of DOTAP is technical, associated with the fabrication of artificial bilayer membranes [29–30].

The lipids are larger molecules than the thiols and present richer spectra, at least in the powder form. The main features in their Raman spectra depend on the hydrocarbon chain, and can be ascribed to scissoring and twisting of CH_2_ and CH_3_ and to stretching of C–C and C–H. More precisely, the bands identified in the higher wavenumber region appear at 3007 cm^−1^ (unsaturated =CH stretching), 2882 cm^−1^ (CH_2_ Fermi resonance) and 2847 cm^−1^ (CH_2_ symmetric stretching). The middle wavenumber region presents bands at 1737 cm^−1^ (C=O ester stretching), 1657 cm^−1^ (C=C stretching), 1442 cm^−1^ (CH_2_ scissoring), 1300 cm^−1^ (CH_2_ twisting) and 1267 cm^−1^ (=C–H in-plane deformation). In the lower wavenumber region, the C–C stretching emerges as a broad band around 1090 cm^−1^. In particular, two contributions at 1065 and 1089 cm^−1^ appear with a shoulder at 1125 cm^−1^. Additional bands appear at 719 and 876 cm^−1^ that are ascribed to the symmetric and asymmetric stretching of choline N^+^(CH_3_)_3_, respectively [31–32].

**Figure 4:**
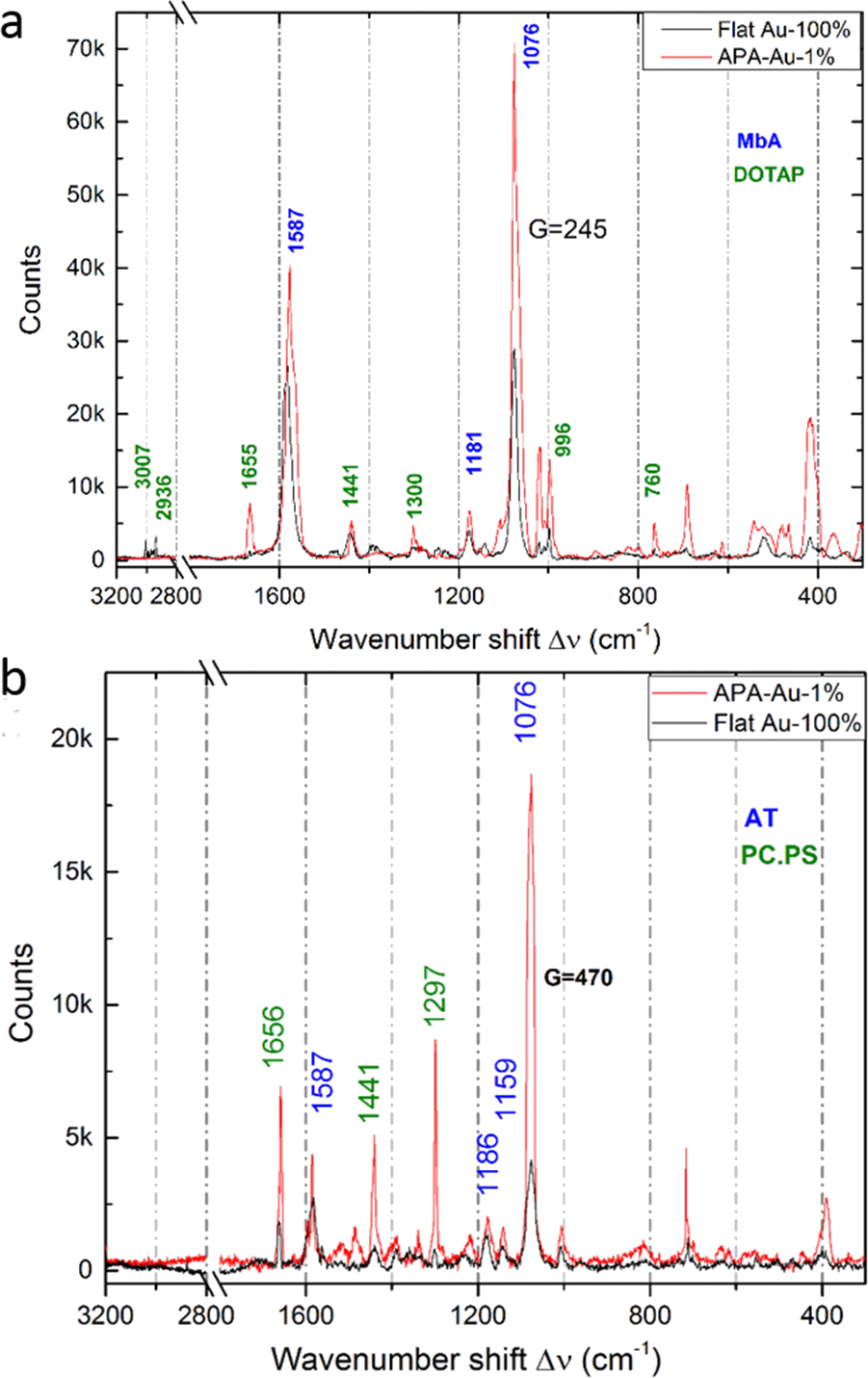
Raman spectra of the thiol-SLB systems on both flat Au and tAPA–Au: a) MbA and DOTAP, b) AT and POPC/POPS blend.

The lipids were further investigated on tAPA–Au substrates, for the possible occurrence of SERS. Figure 4 shows Raman spectra for three mixtures of lipids in SLBs form, on both flat Au and tAPA–Au.

From the comparison of the spectra of lipids in SLBs form versus those in powder form, the most interesting difference observed is that in the films several peaks disappear or are weaker. Whereas some form of quenching can’t be excluded, this is probably due to light polarization constraints in the ordered geometry of the molecular film, where not all modes of chemical groups may be excited, as it can be instead in the assembly of randomly oriented microcrystals of the powders [33].

It appears that on tAPA–Au as compared to flat Au the characteristic thiol peaks are still present and enhanced. As a consequence, for MbA the major peaks at ≈1590 and ≈1080 cm^−1^, ascribed to aromatic ring vibrations, and ≈1181 cm^−1^, ascribed to C–H deformation, appear. Also AT presents the major peaks at ≈1580, ≈1159 and ≈1074 cm^−1^, due to C–NH, N–H wagging and C–C stretching mode, respectively.

Additionally, in Figure 4 we have bands from the lipids, namely ≈1656 cm^−1^ (C=C stretching), ≈1440 cm^−1^ (CH_2_ scissoring), ≈1300 cm^−1^ (CH_2_ twisting), ≈1267 cm^−1^ (=C–H in-plane deformation), and ≈719 cm^−1^ (choline) [34].

The values of enhancement G due to the tAPA–Au nanostructured substrates have been calculated according to Equation 1. Since the peaks on tAPA–Au are 2–4 times higher in the presence of 100 times lower laser power, a G of ≈250 at ≈1076 cm^−1^ and 500 at ≈1076 cm^−1^ is obtained for the thiol–lipid system of MbA–DOTAP and AT–POPC/POPS, respectively.

### QCM-D measurements

The lipid adsorption process on Au was independently monitored by QCM-D technique. This method allows the quantification of the adsorbed mass onto the surface of a vibrating Au-coated quartz electrode through the measurement of the mass-induced frequency shift. Additionally, the measurement of the dissipation gives indication about the viscoelastic properties of the adsorbed layer. The quartz–Au substrate was thus used as a control for success of the incubation of the tAPA–Au substrates in the lipid dispersion. In a preliminary step (data not shown) we have monitored the chemisorption of thiols onto the Au-coated QCM-D sensors; the functionalized sensors where then exposed to the lipid vesicles and the process of adsorption was monitored.

**Figure 5:**
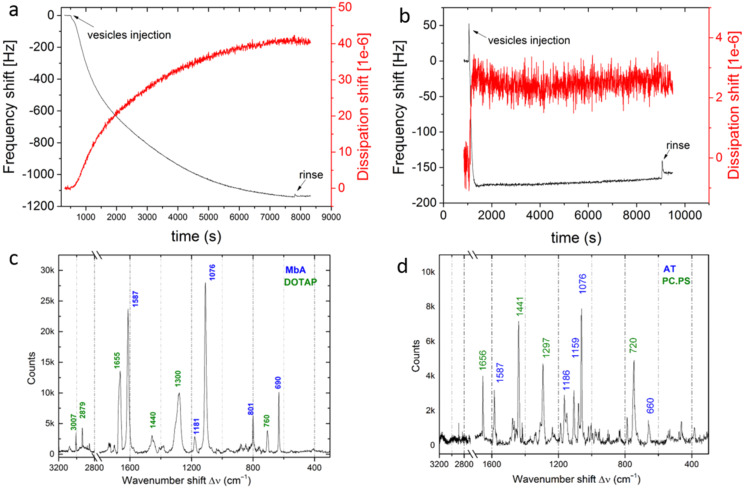
a,b) QCM-D measurements of shift in frequency and dissipation of a) DOTAP on MbA substrate, and b) POPC/POPS on AT substrate; c,d) respective Raman spectra on QCM sensors coated with c) MbA and DOTAP, and d) AT and POPC/POPS blend.

The QCM-D time-evolution profiles presented in Figure 5a,b show that the lipids successfully adsorbed to the Au surface of quartz in both cases. However, the two lipid systems behave differently. For DOTAP on MbA (Figure 5a) one can observe a big shift in frequency (Δ*f* ≈ −1135 Hz) and a high value of dissipation (*D* ≈ 40 × 10^−6^), indicating the adsorption on the sensor’s surface of a viscoelastic structure [35]. DOTAP vesicles do not fuse on Au functionalized with MbA, rather entire vesicles are adsorbed instead. On the contrary, for POPC/POPS on AT (Figure 5b) the frequency shift is low (Δ*f* ≈ −157 Hz for the reported 7th harmonic) and the value of dissipation is close to zero, indicating the adsorption of a smaller mass with more rigid structure on the surface. The reason may be that the POPC/POPS vesicles rupture in contact with the AT-functionalized Au and an SLB forms on the surface [36–37]. Table 1 shows the thickness values of SAM and adsorbed layers obtained with the QCM software. The values have been retrieved by using the Sauerbrey model of rigid layers for the SLBs and the Voigt model of viscoelastic layers for the adsorbed vesicles, assuming for the material densities the following values: ρ_AT_ = 0.9 g/cm^3^ for AT, ρ_MbA_ = 1.34 g/cm^3^ for MbA, and ρ_v_ = 1 g/cm^3^ for vesicles (made mostly of water), according to references [38–39]. The data confirm that DOTAP vesicles adsorb on the sensor without rupturing, with a thickness of the adsorbed layer of ≈90 nm. POPC/POPS vesicles create an SLB on the sensor with a thickness of ≈4 nm. When the formation of an SLB occurs, the fingerprint region is not visible. As already pointed out in Figure 4, we ascribe this effect to the orientation of the molecules and the polarization of the incoming beam. When vesicles are adsorbed on the surface, all the characteristic peaks of the lipid molecules are expected from the Raman spectra, since the vesicles contain all molecular orientations. In accordance to this, the Raman spectra of DOTAP collected from QCM sensor show a signal in the lipid fingerprint region which is different from the spectra collected on the Au–tAPA surface that are flat in the 2800–3000 cm^−1^ region. This indicates that the porosity of the substrate may influence the vesicle fusion process. This finding is still under further investigation.

**Table 1:** Shift in frequency Δ*f* and shift in dissipation *D* and relative standard deviations of the layers adsorbed on the QCM sensor’s surface. Every experiment was repeated three times.

Solution	*d* [nm]	std dev [nm]	Δ*f* [Hz]	std dev [Hz]	*D* [10^−6^]	std dev [10^−6^]

POPC/POPS (milli-Q) AT	3.9	0.1	−92	14	1.5	0.4
DOTAP (PBS) MbA	96	3.5	−1054	19.4	41.5	0.2

In Supporting Information File, Figure S1 the spectra of both lipids are presented again, grouped according to the different types of substrates, which makes it possible to compare the effect of the substrate on the resulting spectra.

## Conclusion

We successfully fabricated tAPA substrates on silicon wafer through anodization of ≈500 nm thickness and post-production etching, resulting in oxide films with pores of ≈160 nm size and ≈80 nm wall thickness. After coating with a ≈25 nm Au layer covering the tAPA features, our substrates become SERS-active and allow for an investigation of the chemical vibrations of molecules, as demonstrated by sensitive Raman measurements on bare thiols and on their combinations with lipid membranes, namely MbA with DOTAP and AT with POPC/POPS. The enhancement factor was estimated to be 500 to 1000 on tAPA–Au with respect to the flat Au surface and to the silicon substrate. The chemisorption of thiols and lipids was confirmed on quartz-Au by QCM-D technique. The present results point to the possible future use of the tAPA–Au surfaces as disposable substrates for assessing the cell functionality in biosensors/bioassays.

## Supporting Information

File 1 Additional figures.
