# Supplementary material for: Surface-enhanced Raman scattering of self-assembled thiol monolayers and supported lipid membranes on thin anodic porous alumina
Source: Beilstein J Nanotechnol. 2017 Jan 9;8:74–81. doi: 10.3762/bjnano.8.8 (PMC5238693; doi:10.3762/bjnano.8.8)
Supplement: File 1 — Additional figures. [file Beilstein_J_Nanotechnol-08-74-s001.pdf]

Supporting Information

for

Surface-enhanced Raman scattering of self-assembled thiol monolayers and supported lipid membranes on thin anodic porous alumina

Marco Salerno[1*], Amirreza Shayganpour[1,2], Barbara Salis[1,2] and Silvia Dante[1]

Address: [1]Department of Nanophysics, Istituto Italiano di Tecnologia, via Morego 30, I-16163 Genova, Italy and [2]Department of Bioengineering and Robotics, University of Genova, viale Causa 13, I-16145 Genova, Italy

Email: Marco Salerno - marco.salerno@iit.it

* Corresponding author

Additional figures

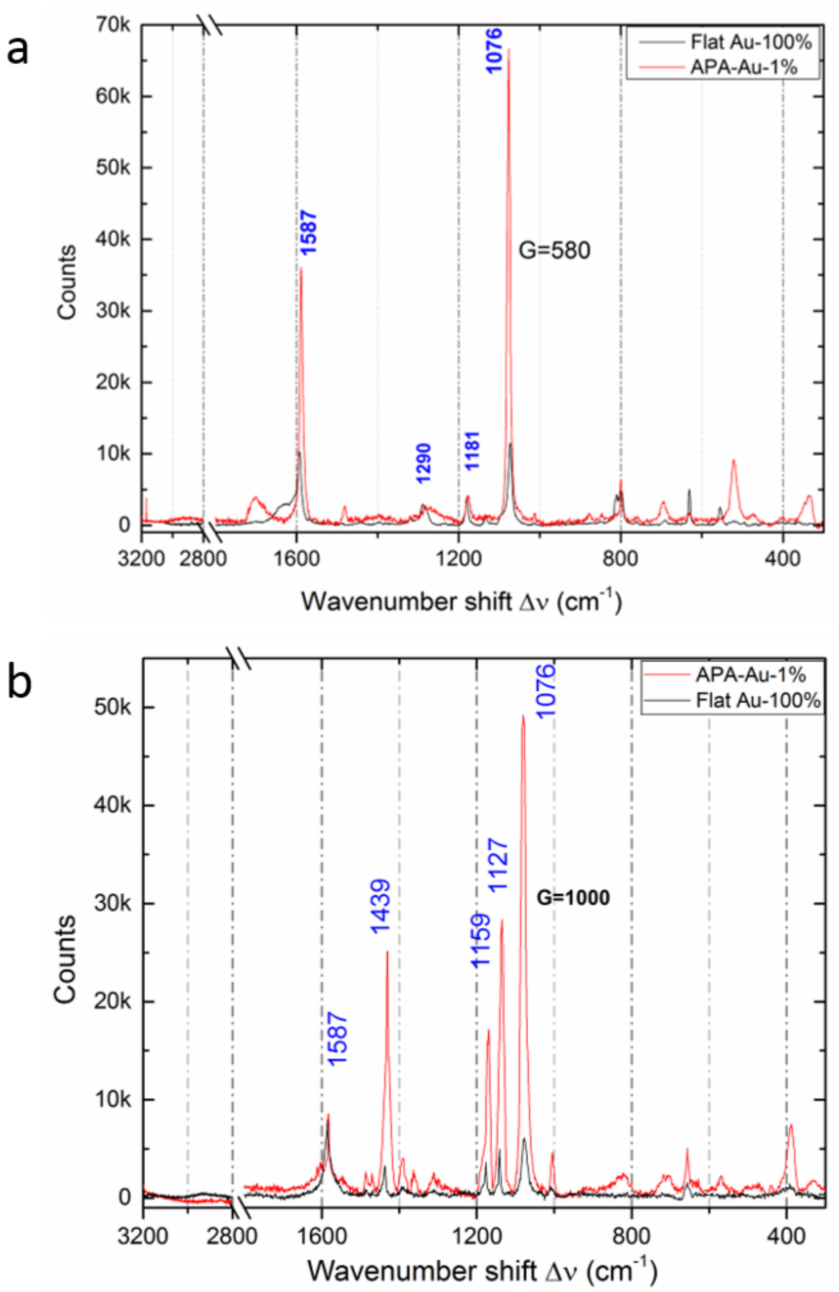

Figure S1: Comparison of Raman spectra of thiols after adsorption to either flat Au or tAPA–Au substrates: a) MbA, and b) AT. The enhancement is appearing clearly (consider also the different laser power, see inset legend).

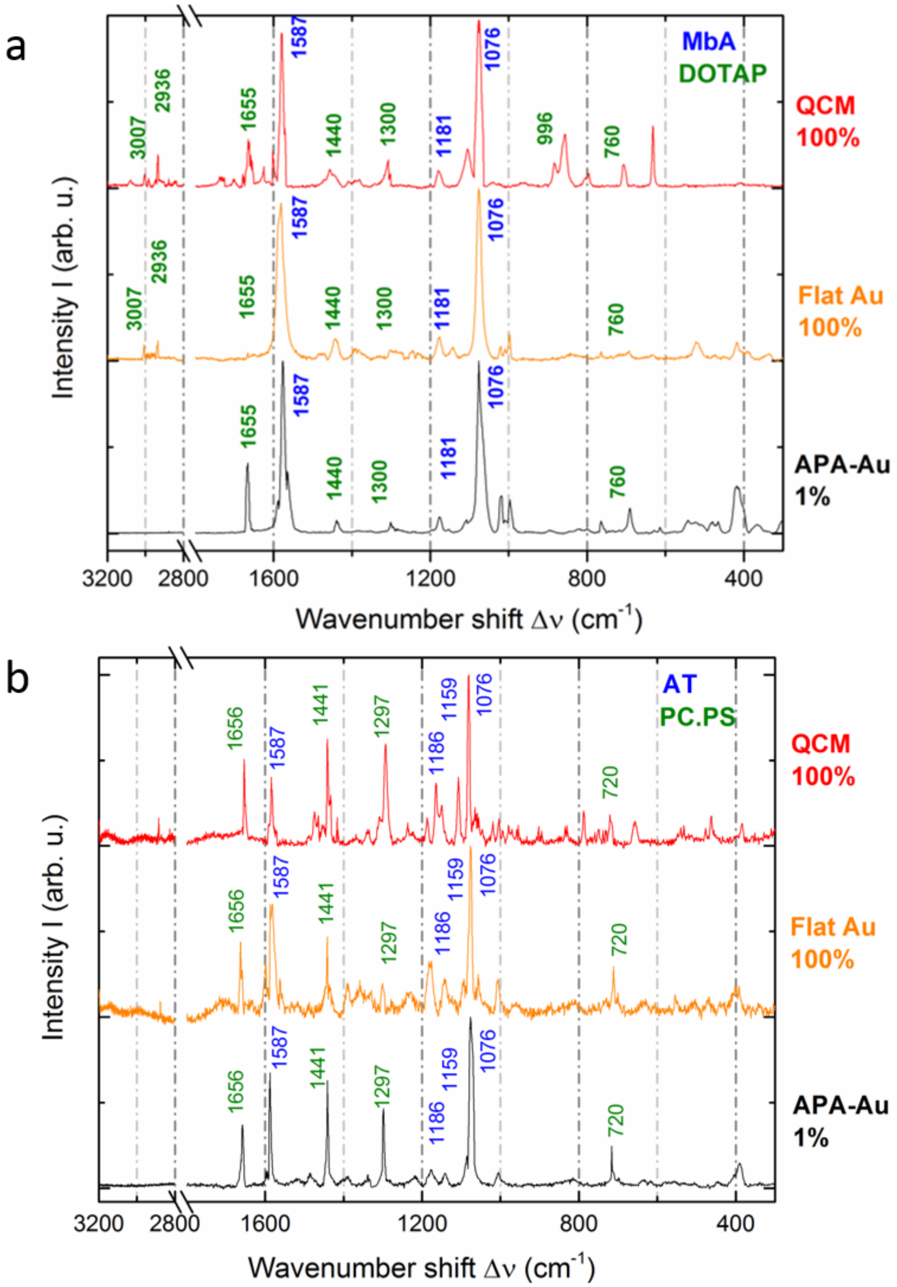

Figure S2: Raman spectra of the thiol-SLB systems on all the different substrates considered in this work, namely flat Au, quartz-Au, and tAPA–Au. a) MbA–DOTAP, b) AT-POPC/POPS, plotted together for easiest comparison of the respective features.
